# Increasing storage stability of freeze-dried plasma using trehalose

**DOI:** 10.1371/journal.pone.0234502

**Published:** 2020-06-11

**Authors:** Raffaele Brogna, Harriëtte Oldenhof, Harald Sieme, Constança Figueiredo, Tobias Kerrinnes, Willem F. Wolkers

**Affiliations:** 1 Unit for Reproductive Medicine—Clinic for Horses, University of Veterinary Medicine Hannover, Hannover, Germany; 2 Biostabilization laboratory—Lower Saxony Centre for Biomedical Engineering, Implant Research and Development, University of Veterinary Medicine Hannover, Hannover, Germany; 3 Institute for Transfusion Medicine, Hannover Medical School, Hannover, Germany; 4 Helmholtz Centre for Infection Research, Braunschweig, Germany; University of Maryland, UNITED STATES

## Abstract

Preservation of blood plasma in the dried state would facilitate long-term storage and transport at ambient temperatures, without the need of to use liquid nitrogen tanks or freezers. The aim of this study was to investigate the feasibility of dry preservation of human plasma, using sugars as lyoprotectants, and evaluate macromolecular stability of plasma components during storage. Blood plasma from healthy donors was freeze dried using 0−10% glucose, sucrose, or trehalose, and stored at various temperatures. Differential scanning calorimetry was used to measure the glass transition temperatures of freeze-dried samples. Protein aggregation, the overall protein secondary structure, and oxidative damage were studied under different storage conditions. Differential scanning calorimetry measurements showed that plasma freeze-dried with glucose, sucrose and trehalose have glass transition temperatures of respectively 72±3.4°C, 46±11°C, 15±2.4°C. It was found that sugars diminish freeze-drying induced protein aggregation in a dose-dependent manner, and that a 10% (w/v) sugar concentration almost entirely prevents protein aggregation. Protein aggregation after rehydration coincided with relatively high contents of β-sheet structures in the dried state. Trehalose reduced the rate of protein aggregation during storage at elevated temperatures, and plasma that is freeze- dried plasma with trehalose showed a reduced accumulation of reactive oxygen species and protein oxidation products during storage. In conclusion, freeze-drying plasma with trehalose provides an attractive alternative to traditional cryogenic preservation.

## Introduction

Human plasma is used for treatment of diseases and diagnostics. Plasma contains coagulation factors (e.g. factor VIII, factor IX), albumin, and immunoglobulins, and can be used to administer missing blood components in patients **[1]**. Different types of diagnostic analyses that can be performed on plasma samples include screening of protein biomarkers (i.e. apolipoproteins and glycoproteins) and assessment of plasma or serum immunoglobulin G (IgG) content which is associated with specific diseases **[2,3,4]**.

If plasma is stored at −20°C for more than 7 days, samples exhibit protein aggregation, and increased proline and glucose contents, which is mainly due to oxidation and acid-base driven hydrolyses reactions as well as enzymatic activities causing changes in plasma metabolite concentrations **[5]**. Therefore, plasma samples should preferably be stored at −80°C **[6]**, where molecular mobility and damaging reactions are drastically slowed down. No degradation of plasma proteins has been reported in plasma samples stored at −80°C or in liquid nitrogen for up to 6 years **[7]**.

Storage of human plasma in the dried state, would allow long-term storage under ambient conditions (i.e. at room temperature), providing an interesting alternative approach for cryogenic preservation. Besides reducing the costs and carbon footprint associated with storage in liquid nitrogen, storage in the dried state can be used in non-laboratory settings where cryogenic storage is not an option (e.g. non-hospital settings, battlefield medicine, and in underdeveloped countries or areas with limited infrastructures).

Human plasma preserved in a dried state, first appeared in the medical literature in the 1930s, and was used by American armed forces in World War II and in the Korean War **[8]**. However, many cases of hepatitis transmission have led to a temporary stop in the use of freeze-dried plasma. This was not related to the drying procedure per se, but to the risk of pathogen transmission when using pooled plasma products **[9]**. Pathogen reduction methods dramatically improved the safety profiles, and dried plasma is currently used by the French Military and the German Red Cross for both military and civilian emergency medical applications **[8]**. When freeze-dried plasma is analyzed after long-term storage under different conditions, levels of clotting factors (except for factor V and INR) do not exceed standard range values for the duration of its shelf life **[10]**. However, many clinical trials aiming to investigate feasibility of dried plasma are still in process, including regulatory pathway, logistical and product issues **[11]**. Preclinical investigation of dried plasma in hemorrhagic shock and traumatic endotheliopathy models, support the needs of future studies for dried plasma **[12]**.

Exposure of biological specimens to freezing and/or drying may result in drastic changes in their chemical and physical properties **[13,14]**. Molecular interactions typically change during lowering the temperature and removal of bound water, resulting in biomolecular phase and structural changes as well as aggregation **[15]**. In addition, reactive oxygen species (ROS) such as superoxide anion radicals accumulate, which in turn may react with biomolecules (i.e. lipids, proteins, nucleic acids) therewith impairing their function and recognition sites **[16]**. Oxidative damage of proteins results in formation of protein carbonyl groups, and assessment of their content can be used as a marker of overall protein oxidation **[17]**. Freeze-drying requires specific protective agents, referred to as lyoprotective agents. The disaccharides sucrose and trehalose, which can be found in high concentrations in anhydrobiotic organisms **[18,19]**, function as osmoprotectants during freezing, replace water that surrounds biomolecules during drying, and facilitate the formation of a protective glassy state **[14,20]**.

Stabilizing excipients for freeze-dried plasma such as sucrose, trehalose, sorbitol, mannitol and glycine have been evaluated under accelerated aging storage conditions **[21]**. Interestingly, glycine outperformed the sugars in this study, but only one concentration was tested. Other studies based on of C4B peptide cleavage, where no stabilizing excipients were used, showed that freeze-drying followed by room temperature storage, was equally effective in preventing plasma protein degradation compared to frozen storage at −80°C, for up to 1 year **[22]**. However, pre-lyophilization processing (e.g. use of protease inhibitors) is necessary to avoid C4B degradation, so this study does not accurately reflect the stability of most plasma proteins. Dried plasma specimens have been successfully used for immunodetection of hepatitis **[23]** and quantification of HIV coding ribonucleic acids **[24]**. However, little is known about the storage stability of plasma proteins and other components during long-term storage in the dried state **[9]**.

The aim of this study was to investigate the possible beneficial effects of using sugars (glucose, sucrose, trehalose) as protectants for freeze-drying of blood plasma. Differential scanning calorimetry was used to determine glass transition temperatures of freeze-dried samples. The extent of protein aggregation before and after freeze-drying, storage and rehydration was evaluated from turbidity measurements, and the protein secondary structure was evaluated using Fourier transform infrared spectroscopy (FTIR) combined with principal component analysis (PCA). In addition, oxidative damage (i.e. accumulation of reactive oxygen species and protein carbonyl groups) was studied in plasma samples with/out trehalose, and during hydrated/frozen/dried storage at different temperatures.

## Materials and methods

### Plasma samples, freeze-drying and dry storage conditions

Plasma was provided by the Institute for Transfusion Medicine of the Hannover Medical School. Human plasma was obtained from healthy volunteers with informed consent. Use of human blood was carried out after ethical approval according to legal provisions and rules of the Hannover Medical School (Ethics Committee of Hannover Medical School (MHH)). Plasma was prepared from whole blood donations using heparin as anticoagulant. Blood was mixed with saline-adenine-glucose-mannitol (SAGM) as preservative solution. Plasma was obtained by centrifugation of anti-coagulated whole blood and taking the supernatant as plasma fraction.

For studies on pure immunoglobulin G (IgG), human IgG was purchased (Lee Biosolutions Inc) as freeze-dried powder and reconstituted according to instructions provided by the manufacturer; in phosphate buffered saline (PBS), at 10 mg mL^−1^.

Prior to use, frozen plasma aliquots (50 mL) were thawed by incubation for 30 min at 37°C, where after further handling was done at room temperature. Plasma was divided into smaller samples for adding protective agents, which included glucose (Sigma-Aldrich), sucrose (Roth) and trehalose (Cargill). Two-fold strength protective formulations [0/5/10/20% (w/v) sugar] were prepared in water to obtain final concentrations up to 10% (w/v) after mixing 1/1 (v/v) with plasma. Freeze-drying of 1 mL samples was done in 2R injection glass vials, using a lyophilizer with temperature-controlled shelves (Virtis Advantage Plus Benchtop freeze dryer; SP scientific). Samples were cooled from room temperature down to −30°C at 1°C min^−1^, after which specimens were kept at −30°C for 2 h. Primary drying was performed for 5 h at a temperature of −30°C and a pressure of 60 mTorr. For secondary drying, the shelf temperature was increased to 40°C at 0.1°C min^−1^ and samples were maintained at 40°C and 60 mTorr for 12 h, after which the temperature was reduced and kept at 20°C until samples were taken out. After lyophilization, samples were either directly analyzed or stored under different conditions. Samples were rehydrated by adding 1 mL water. Freeze-dried samples were vacuum-sealed to prevent moisture uptake during storage; and stored in darkness for up to 40 days at different temperatures.

### Turbidity measurements for simple analysis of protein structure

Freeze-drying induced protein aggregation tends to increase sample turbidity. This can be analyzed by measuring the absorbance at a specific wavenumber as a measure for the extent of light scattering. Absorbance measurements, for hydrated, freeze-dried and stored plasma and IgG samples were done using a UV/VIS Libra S60PC spectrophotometer (Biochrom Inc) and Nanodrop 2000 spectrophotometer (Thermofisher Scientific), respectively, at 550 and 350 nm as described elsewhere **[25,26]**. Freeze-dried samples were rehydrated just prior to analysis.

### Analysis of accumulation of oxidative damage, using nitroblue tetrazolium

Accumulation of reactive oxygen species (ROS) during storage of plasma samples was analyzed using nitroblue tetrazolium (NBT; Roth). Upon reaction with ROS, formazan is formed which is visible as a blue coloration and can be quantified spectrophotometrically. NBT (10 mg mL^‒1^) was first dissolved in PBS (at room temperature for 1 h, while stirring/mixing), after which the solution was passed through a 0.2-μm filter for removing particles. NBT was added to plasma samples, prior to freeze-drying. This was done by adding together: 0.250 mL plasma, 0.125 mL freshly prepared NBT solution and 0.125 mL water or four-fold lyoprotectant formulation; resulting in a final sample volume of 0.5 mL, up to 10% (w/v) sugar, and 2.5 mg mL^−1^ NBT. To quantify formazan production, after rehydration with 0.5 mL water, samples were incubated for a defined duration (30 min at 37°C), after which formazan was solubilized in dimethyl sulfoxide (0.5 mL) and samples were centrifuged to remove debris. Absorbance values at 530 nm were measured spectrophotometrically **[27]**.

### Determination of the total protein and protein carbonyl content

Plasma total protein contents were determined using a commercially available bicinchoninic acid (BCA) assay; according to the instructions provided by the manufacturer (Pierce). In short, 50 μL plasma sample with(out) trehalose was added to 200 μL freshly prepared BCA working reagent [composed of reagent A and B, 50/1 (v/v)]. Samples were incubated for 30 min at 37°C, after which the absorbance was measured spectrophotometrically at 570 nm. BSA was used for preparing a standard curve (0−2 mg mL^−1^).

Plasma protein carbonyl contents were analyzed using previously described **[28]**, using a commercially available kit (Sigma-Aldrich). With this assay, protein carbonyl groups reacting with added 2,4-dinitrophenylhydrazine (DNPH) will give rise to the formation of dinitrophenyl hydrazine adducts, which can be quantified spectrophotometrically at 375 nm. Briefly, plasma samples (100 μL) were mixed with DNPH solution (100 μL). After 10 min incubation at room temperature, trichloroacetic acid (30 μL) was added, samples were vortexed, centrifuged (13,000×g, 2 min), and the pellet with precipitated proteins was washed with ice-cold acetone (500 μL). Then, 200 μL 6 M guanidinium hydrochloride was added and samples incubated at 60°C (10 min) until the pellet was dissolved. Absorbance values at 375 nm were recorded and the carbonyl content could be calculated using the molar absorption coefficient (22,000 M^−1^ cm^−1^). Values were expressed with respect to the total protein content of the sample.

### Determination of the plasma IgG

The total IgG content of plasma samples was determined using a commercially available enzyme linked immunosorbent assay (ELISA) kit (Novateinbio); according to the instructions provided by the manufacturer. This kit consists of a 96 well microplate coated with an antibody against human IgG. In short, plasma samples (100 μL) were added in the wells and incubated for 2 h at room temperature. The plate was washed (3×), followed by 2 h incubation with anti-IgG antibody. After washing (3×), streptavidin-HRP solution was added followed by a 30 min incubation. Finally, after washing (3×), 3,3′,5,5′-tetramethylbenzidine (TMB) substrate solution was added and the change in absorbance at 405 nm was measured after 15 min, using a microplate reader (BioTek Instruments Inc). IgG (see above) was used for preparing a standard curve (0−80 μg mL^−1^).

### Analysis of protein secondary structure using Fourier transform infrared spectroscopy

The overall protein secondary structure of plasma samples freeze-dried with different sugars was studied using Fourier transform infrared spectroscopy (FTIR). Infrared spectra were recorded using a Nicolet iS5 FTIR spectrometer (Thermo-Fisher), equipped with a triglycine sulfate detector and an attenuated total reflection (ATR) accessory, with pressure arm and a diamond/ZnSe crystal. Dry material was pressed on the ATR-crystal using similar conditions for all samples tested. Spectra acquisition parameters were: 4 cm^−1^ resolution, 8 co-added interferograms, 4000−650 cm^−1^ wavenumber range, and an automatic CO_2_/H_2_O vapor correction algorithm. Spectra analysis was done using Omnic software (Thermo-Fisher). Freeze-dried specimens were analyzed directly after the freeze-drying process as well as after storage at different temperatures and/or for different durations.

In order to evaluate the overall protein secondary structure, the 1700–1600 cm^−1^ spectral region, containing the amide-I absorbance band, was selected and normalized. Second derivative spectra were processed with a 21-point smoothing factor, using the Savitzky-Golay method. This was done to better resolve the absorbance bands representing α-helical and β-sheet structures at ~1650 and ~1630 cm^‒1^, respectively. Differences amongst samples were quantified by calculating the ratio of the intensities of these bands [i.e. I(ν1630)/I(ν1650)].

In addition, principal component analysis (PCA) was used to analyze FTIR spectra obtained from the different treatment groups. PCA allows assessing differences among treatment groups, by applying multivariate analysis and reducing the number of variables in a multidimensional dataset **[29]**. The main goal of PCA is to obtain a small set of principal components (PC) that explain the most variability on these data sets. Prior to performing PCA, the protein spectral region between 1700 and 1600 cm^−1^ was selected and subjected to a linear base line correction and vector normalization. Vector normalization is carried out in the following way: spectra are first mean-centred, i.e. the average value of the absorbances is calculated for the selected spectral region. This value is then subtracted from the spectrum. Then, the spectra are scaled such, that the sum squared deviation over the indicated wavelengths equals one:

$$a_{m}=\frac{\sum_{k}a(k)}{N(k)}$$ (1)

$$a´(k)=a(k)-a_{m}$$ (2)

$$a^{´´}(k)=\frac{a^{´}(k)}{\sqrt{\sum_{k}(a^{´}{\left(k)\right)}^{2}}}$$ (3)

$$\sum_{k}(a^{´´}{\left(k)\right)}^{2}=1$$ (4)

where a(k) reflects the spectral intensity at wavenumber k, and N(k) the total number of discrete wavenumbers in the selected spectral region. PCA was performed using MATLAB (Mathworks). Plots were constructed in which principal component (PC) 1 was plotted versus PC2, to graphically visualize differences among treatment-groups.

### Thermal analysis of freeze-dried plasma samples using differential scanning calorimetry

Differential scanning calorimetry (DSC) measurements were carried out using a Netzsch DSC 204F1 Phoenix instrument (Netzsch Geraetebau GmbH). Calibration was performed using adamantane, bismuth, indium, zinc, selenium, and cesium chloride, according to the instructions provided by the manufacturer. An empty pan was used as a reference sample. For analysis of freeze-dried plasma with(out) lyoprotectants, 10 mg material was added into a 25-μL aluminum DSC pan. The sample weight was determined using a microbalance, and pans were sealed hermetically. Samples were cooled from 20°C down to ‒30°C followed by heating to 120°C, both at 10°C min^−1^, while monitoring the heat flow. Samples were exposed to cooling-and-heating twice, and held at ‒30°C and 120°C for 10 min. The first scan was used to obtain a uniform sample, whereas glass transition temperatures (Tg-values) were determined from the second heating scan. DSC thermograms were analyzed using Proteus thermal analysis software (Netzsch Geraetebau GmbH), and the Tg was determined as midpoint of the temperature range in which the glass transition occurred.

Sample dry weights were determined after DSC analyses, after piercing pans and overnight incubation in an incubator set at 80°C. The sample water content (WC; in g water per g dry weight) was determined by comparing the original/fresh and dry weight:

$$WC=\frac{\left[(FW+P)-P]-[(DW+P)-P\right]}{\left[(DW+P)-P\right]}$$ (5)

here FW and DW represent respectively the fresh and dry weight of the sample, and P the weight of the DSC pan (all in g).

### Statistical analysis

Experiments were performed at least in triplicate, unless otherwise stated, using different plasma units. Data are reported as means ± standard deviations. To determine if differences amongst sample characteristics are statistically significant (p<0.05), analysis of variance (ANOVA) was used followed by Tukey’s multiple comparisons test.

## Results

### Thermal analysis of freeze-dried plasma samples using differential scanning calorimetry

**Fig 1:**
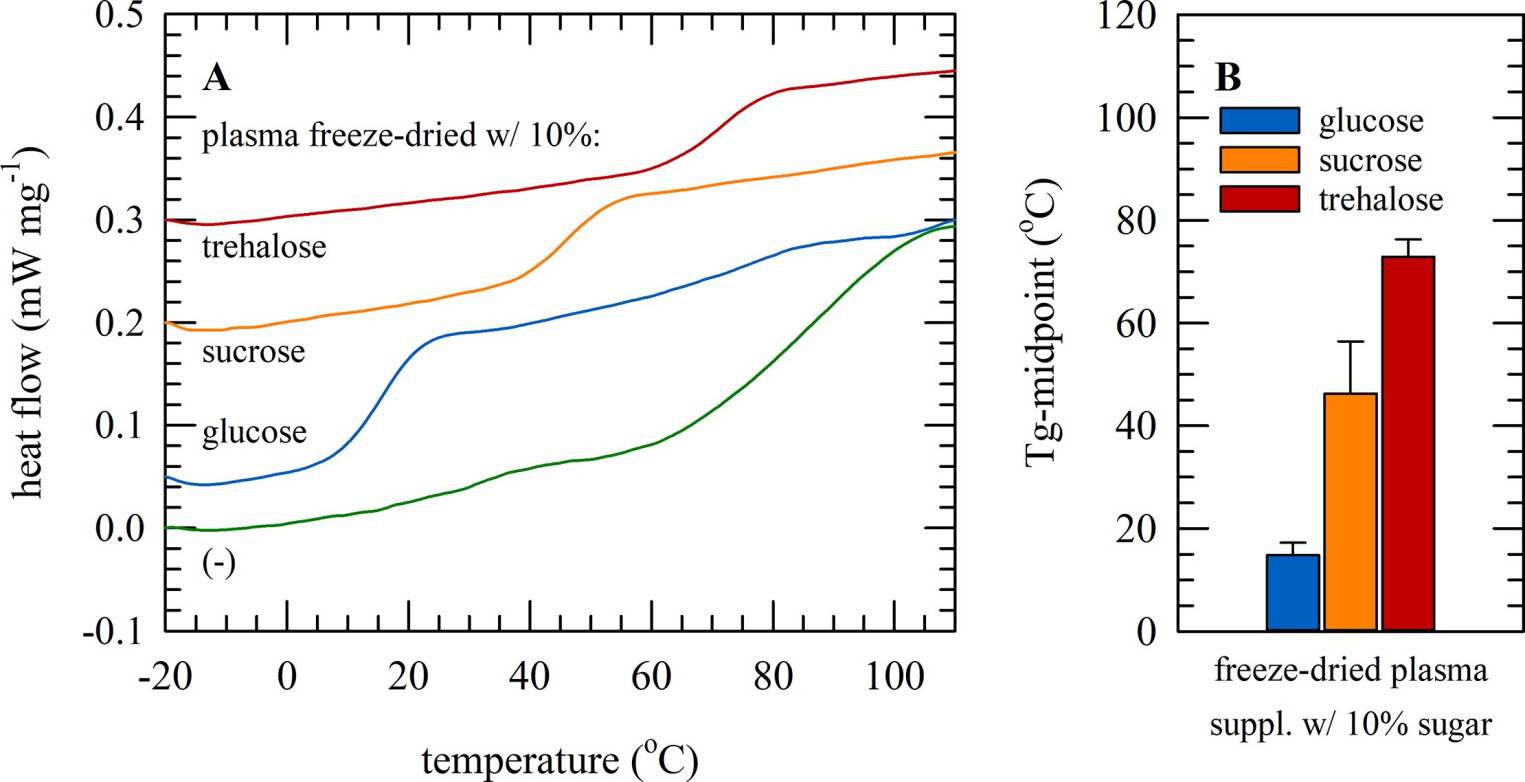
Glass transition temperatures of freeze-dried plasma samples. DSC thermograms of plasma samples that were freeze-dried without protectants (green), or freeze-dried with 10% glucose (blue), sucrose (orange), or trehalose (red). Representative thermograms are shown (A). Tg-values were determined as the midpoint of the temperature ranges were glass transition occurred. Measurements were done in triplicate, and mean values ± standard deviations are presented (B).

Freeze-dried samples had a water content of 0.07±0.01 g H_2_O g DW^−1^. Fig 1 shows DSC thermograms of plasma freeze-dried without and with 10% (w/v) sugar. Tg-values were determined as the midpoint temperature at which the change in specific heat coinciding with the transition from glassy-to-liquid state occurred. It was determined that plasma samples freeze-dried with trehalose exhibited the highest Tg (72±3.4°C) followed by sucrose (46±11°C) and glucose (15±2.4°C). This implies that plasma samples freeze-dried with trehalose or sucrose will be in a glassy state if stored under ambient conditions. For trehalose, there is a greater difference between the storage temperature and Tg, which could be beneficial for storage under suboptimal conditions. Therefore, trehalose was selected for further studies on storage stability of plasma samples.

### Freeze-drying-induced protein aggregation is prevented if sugars are added to plasma

**Fig 2:**
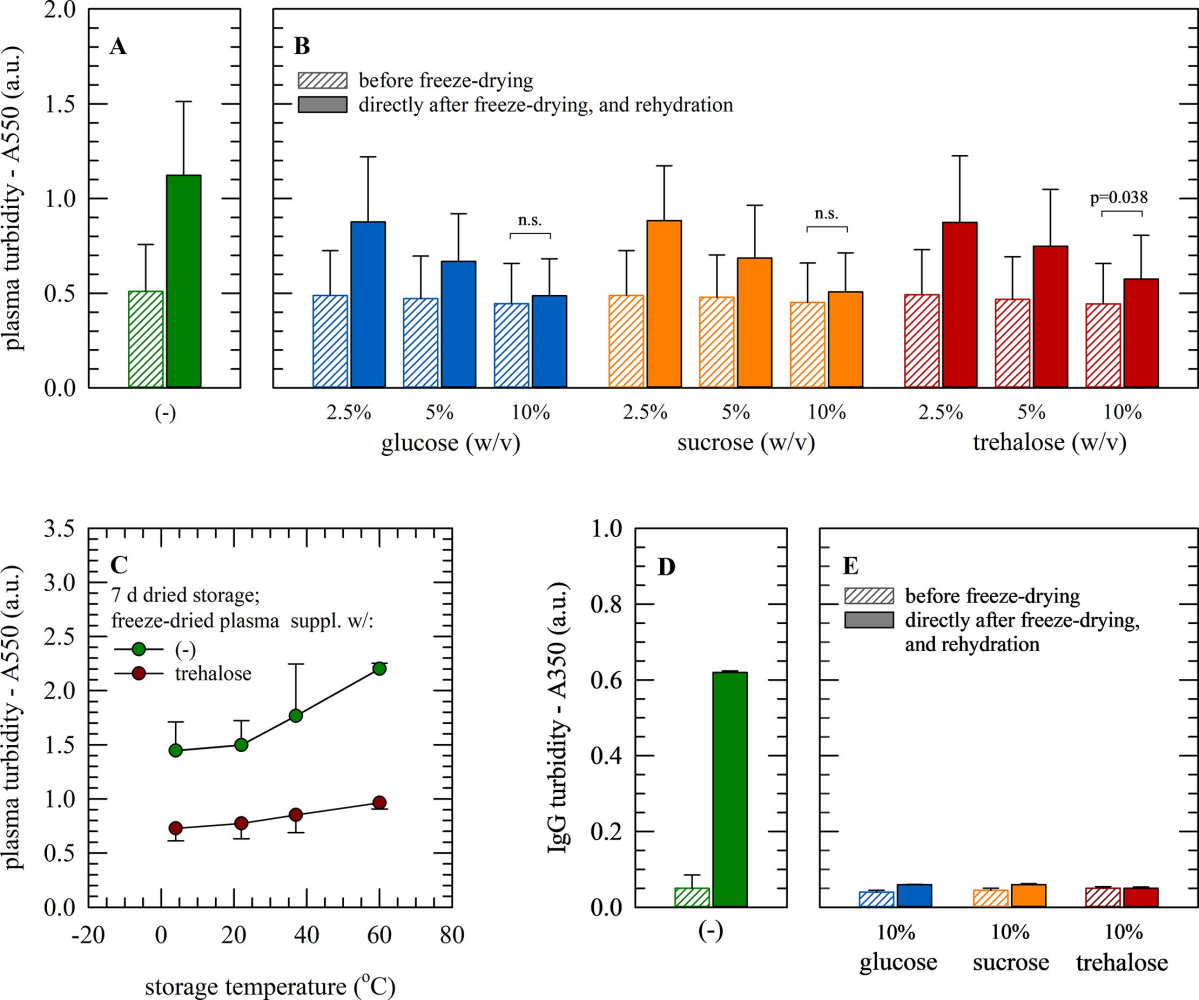
Turbidity of plasma after freeze-drying and rehydration. Human plasma (A−C) and pure IgG (D,E) were freeze-dried without (green) protectants as well as 2.5−10% glucose (blue), sucrose (orange) or trehalose (red). Samples were analyzed before (bars with diagonals) and directly after (filled bars) freeze-drying (A,B,D,E) or after 7 d dried storage at temperatures ranging from 4−60°C (C). Protein aggregation was evaluated by means of turbidity as the absorbance at 550 nm (human plasma) or 350 nm (IgG). Mean values ± standard deviations are presented. For plasma, three technical replicates were performed for plasma obtained from six different donors. For IgG, measurements were done in triplicate.

Protein aggregation in human plasma causes a change in sample turbidity, which is visible as an increase in light scattering. It was investigated if freeze-drying increases turbidity (caused by protein aggregation) and if this can be avoided by using lyoprotectants. In Fig 2A and 2B, it can be seen that turbidity of samples freeze-dried without protectant is increased after rehydration; A550-values were increased two-fold as compared to samples not exposed to freeze-drying. Plasma supplemented with sugars prior to freeze-drying, displayed a dose-dependent decrease in turbidity with increasing sugar concentration. Glucose, sucrose and trehalose appear to be equally effective in diminishing protein aggregation, and when using a 10% sugar concentration, plasma turbidity/A550-values were found to be similar compared to those before freeze-drying. Variation amongst plasma units obtained from six different units was investigated using three technical replicates each (Table 1). Significant differences in plasma turbidity were found amongst plasma from different donors prior to freeze-drying, but the trend after freeze-drying and rehydration was the same for all batches. Statistical analysis revealed no significant differences in A550-values before and after freeze-drying when a 10% glucose or sucrose was used, whereas for 10% trehalose there was still a small/significant difference in turbidity seen for 3 out of 6 donors.

**Table 1:** Turbidity of plasma after freeze-drying and rehydration. Spectroscopic analysis of plasma turbidity (i.e. absorbance at 550 nm), both before and after freeze-drying of plasma without supplements (-) as well as supplemented with 2.5−10% glucose (GLU), sucrose (SUC) or trehalose (TRE). Plasma from six different donors was analyzed (L1−6), with performing three technical replicates each. Mean ± standard deviations are presented, while statistically significant (p<0.05) differences amongst the time point of analysis (i.e. pre versus post freeze-drying) are indicated with an asterisk, and those between different sugar concentrations used (post freeze-drying data only) are indicated with different superscript letters. Values between different donors were statistically significant for all cases.

	pre freeze-drying						post freeze-drying					
formulation	L1	L2	L3	L4	L5	L6	L1	L2	L3	L4	L5	L6
**-**	0.782±0.004	0.424±0.004	0.724±0.051	0.375±0.091	0.091±0.002	0.661±0.006	1.732±0.048	1.230±0.113	1.195±0.066	0.958±0.040	0.466±0.019	1.153±0.027
**GLU-2.5%**	0.716±0.003	0.393±0.003	0.733±0.003	0.368±0.007	0.087±0.005	0.631±0.004	1.323^a^±0.015	0.982^a^±0.042	1.032^a^±0.030	0.682^a^±0.009	0.258^a^±0.006	0.983^a^±0.017
**GLU-5%**	0.690±0.01	0.383±0.002	0.695±0.003	0.373±0.005	0.082±0.002	0.609±0.002	0.910^b^±0.057	0.656^b^±0.047	0.866^b^±0.005	0.578^b^±0.019	0.187^a,b^±0.005	0.808^b^±0.063
**GLU-10%**	0.623±0.004	0.361±0.004	0.680±0.008	0.339±0.014	0.080±0.002	0.584±0.004	0.692^c,^*±0.036	0.400^c,^*±0.003	0.634^c,^*±0.007	0.454^c^±0.027	0.128^b,^*±0.004	0.610^c,^*±0.019
**SUC2.5%**	0.723±0.011	0.393±0.004	0.724±0.002	0.362±0.007	0.088±0.004	0.638±0.004	1.240^a^±0.027	1.007^a^±0.036	0.970^a^±0.016	0.686^a^±0.021	0.393^a^±0.214	1.004^a^±0.007
**SUC-5%**	0.682±0.017	0.387±0.003	0.704±0.004	0.383±0.019	0.087±0.004	0.624±0.004	0.999^b^±0.008	0.776^b^±0.049	0.796^b,^*±0.018	0.516^b^±0.021	0.171^b,^*±0.006	0.853^b^±0.016
**SUC-10%**	0.610±0.006	0.361±0.003	0.685±0.007	0.382±0.008	0.086±0.003	0.586±0.005	0.751^c,^*±0.019	0.474^c^±0.011	0.638^c,^*±0.086	0.409^c,^*±0.017	0.141^b,^*±0.020	0.628^c,^*±0.010
**TRE-2.5%**	0.710±0.006	0.393±0.002	0.759±0.004	0.368±0.007	0.092±0.002	0.631±0.004	1.315^a^±0.134	0.995^a^±0.090	0.988^a^±0.025	0.643^a^±0.018	0.259^a^±0.011	1.043^a^±0.025
**TRE-5%**	0.635±0.004	0.386±0.007	0.736±0.005	0.352±0.006	0.085±0.001	0.612±0.005	1.061^a^±0.049	0.890^a^±0.010	0.858^b^±0.025	0.566^a^±0.018	0.189^b^±0.006	0.925^b^±0.011
**TRE-10%**	0.616±0.005	0.362±0.004	0.683±0.005	0.333±0.008	0.082±0.001	0.588±0.006	0.783^b^±0.010	0.574^b^±0.025	0.781^b^±0.018	0.427^b,^*±0.008	0.157^b,^*±0.005	0.726^c,^*±0.022

Evaluation of plasma samples after 7 days dried storage at different temperatures (Fig 2C), revealed that plasma freeze-dried with 10% trehalose, exhibited no changes in turbidity/protein aggregation upon rehydration, irrespective of the storage temperature. In addition to a higher initial value, for plasma freeze-dried without protectants, turbidity upon rehydration increased with increasing storage temperature. It should be noted that for practical reasons frozen plasma was used here, so samples that were used for freeze-drying first underwent a freeze-thaw cycle.

In case of freeze-drying pure IgG, a similar trend was observed as seen for plasma samples. Freeze-drying-induced turbidity/protein aggregation could be prevented by adding 10% sugar (Fig 2F). It should be noted that IgG is sold as a lyophilized powder using salts as excipient, which can also prevent freeze-drying induced IgG aggregation.

### Freeze-drying-induced changes in protein secondary structure can be prevented by sugars

**Fig 3:**
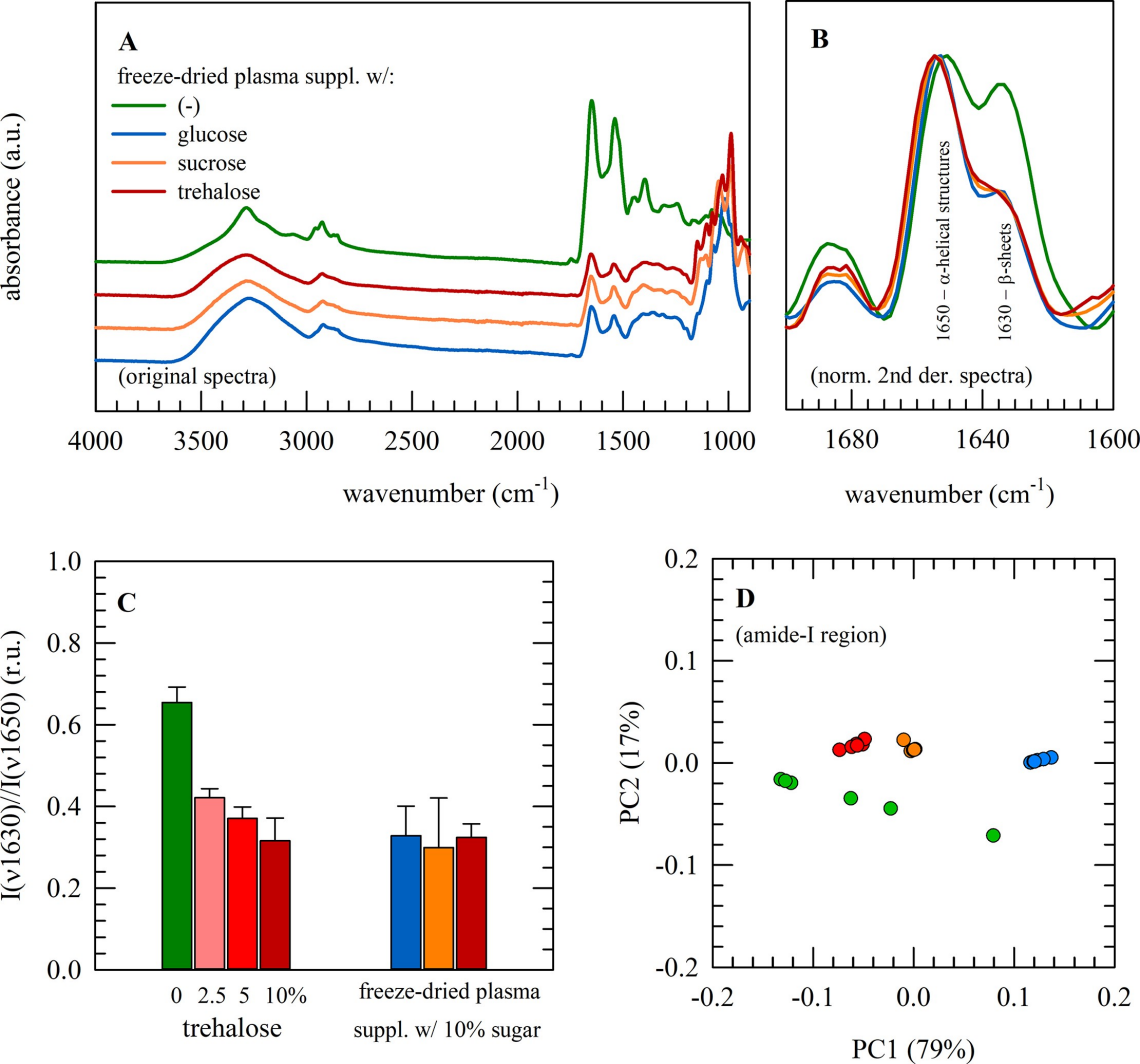
Infrared spectroscopic analysis of freeze-dried plasma. Infrared spectroscopic analysis of plasma freeze-dried without protectants (green), or freeze-dried with glucose (blue), sucrose (orange), or trehalose (red). Full original spectra are presented (A) as well as normalized second derivative spectra of the amide-I protein region (B). To reveal relative contributions of α-helical and β-sheet structures at ~1650 and ~1630 cm^‒1^, respectively, band intensity ratios were calculated; for plasma freeze dried with increasing trehalose concentrations, as well as 10% glucose and sucrose (C). Furthermore, the amide-I protein region (1700−1600 cm^‒1^) was subjected to PCA and scores plots of the first two principal components were prepared (D). Measurements were repeated 3−6 times, and mean values ± standard deviations are presented.

FTIR was used to study the overall protein secondary structure of freeze-dried plasma (Fig 3). In case samples were freeze-dried with sugars, glass formation is evident from the broad shape of the OH-stretching vibration band (3600−3000 cm^−1^). In addition, sugar specific peaks can be found in the fingerprint region ranging from 1500−900 cm^−1^. To reveal possible differences in protein secondary structure amongst samples, the amide-I wavenumber region (1700‒1600 cm^−1^) was inspected. Normalized second derivative spectra were calculated to determine the contributions of α-helical and β-sheet structures, at respectively 1650 and 1630 cm^−1^. In Fig 3B, it can be seen that freeze-dried plasma without protectant exhibits a higher relative content of β-sheet structures as compared to freeze-dried plasma supplemented with 10% sugar. Formation of β-sheet structures, expressed as the β-sheet versus α-helix band intensity ratio I(ν1630)/I(ν1650), decreases with increasing sugar concentration (Fig 3C).

PCA was conducted on the amide-I region to corroborate the differences that can be observed in the spectra (Fig 3D). PCA is used to reveal differences among different treatment groups, which cannot be easily picked up in the raw spectra. PCA reduces the large multidimensional FTIR dataset by geometrically projecting spectral data sets (wavenumber versus intensity plots) into lower dimensions, so called principal components (PCs), to obtain a reduced data set that can be more easily visualized and analyzed. The first principal component (PC1) represents the maximum variance in the data set. Usually, one principal component is insufficient to model the systematic variation of a data set. Thus, a second principal component (PC2), which is orthogonal to PC1, is needed. Score plots, of PC1 versus PC2, can thus be used to reveal spectral differences among treatment groups (i.e. with or without sugars) as they appear in different clusters. PC1 describes most of the observed variance (~79%) and allows a clear discrimination between plasma freeze-dried with and without sugars. Moreover, plasma samples freeze-dried with sugars exhibit more compact score plots, indicating they are more homogeneous.

### Plasma protein aggregation, secondary structure and IgG content during storage

**Fig 4:**
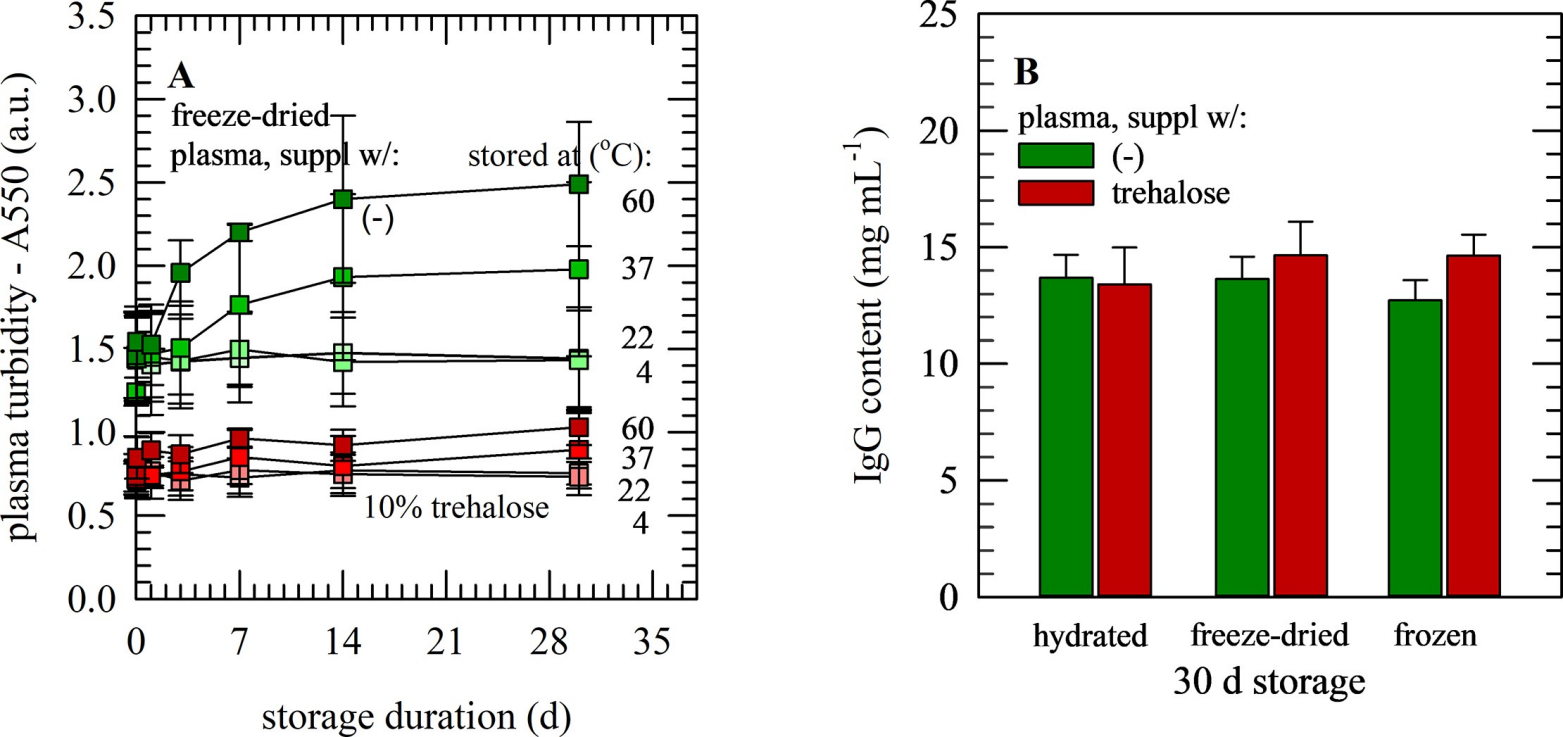
Storage stability of freeze-dried plasma samples. Turbidity/protein aggregation characteristics of freeze-dried plasma samples stored for up to 1 month at temperatures ranging from 4−60°C (A), as well as plasma IgG contents after storage under different conditions (B). Freeze-drying was done without supplements (green) as well as with supplementation of 10% trehalose (red). Turbidity was assayed directly after rehydration, as the absorbance at 550 nm (A). IgG contents were determined for plasma samples after 30 d storage; for hydrated sample and freeze-dried samples stored at 22°C, as well as frozen samples stored at −80°C. Mean values ± standard deviations are presented from replicate measurements performed using plasma from three different donors.

As described above, plasma turbidity increased in samples freeze-dried without protectants, and freeze-drying-induced protein aggregation can almost entirely be prevented if plasma is freeze-dried with 10% trehalose. Turbidity of freeze-dried samples without protectants increases with time and temperature (1.77±0.48 at 37°C and 2.20±0.05 at 60°C versus 1.50±0.23 at room temperature; Fig 4A). In contrast, it was found that the temperature dependent increase in protein aggregation could be prevented if plasma samples were freeze-dried with trehalose. When trehalose was added, there was only a minor increase in turbidity with increasing temperature (0.85±0.16 at 37°C and 0.96±0.06 at 60°C versus 0.77±0.14 at room temperature; Fig 4A). However, no differences in IgG contents were observed. The total IgG content of hydrated and freeze-dried samples, which were stored for 30 d at ambient temperature, were similar as those determined for samples stored frozen at −80°C, and no differences were seen for specimens supplemented with(out) trehalose (Fig 4B).

**Fig 5:**
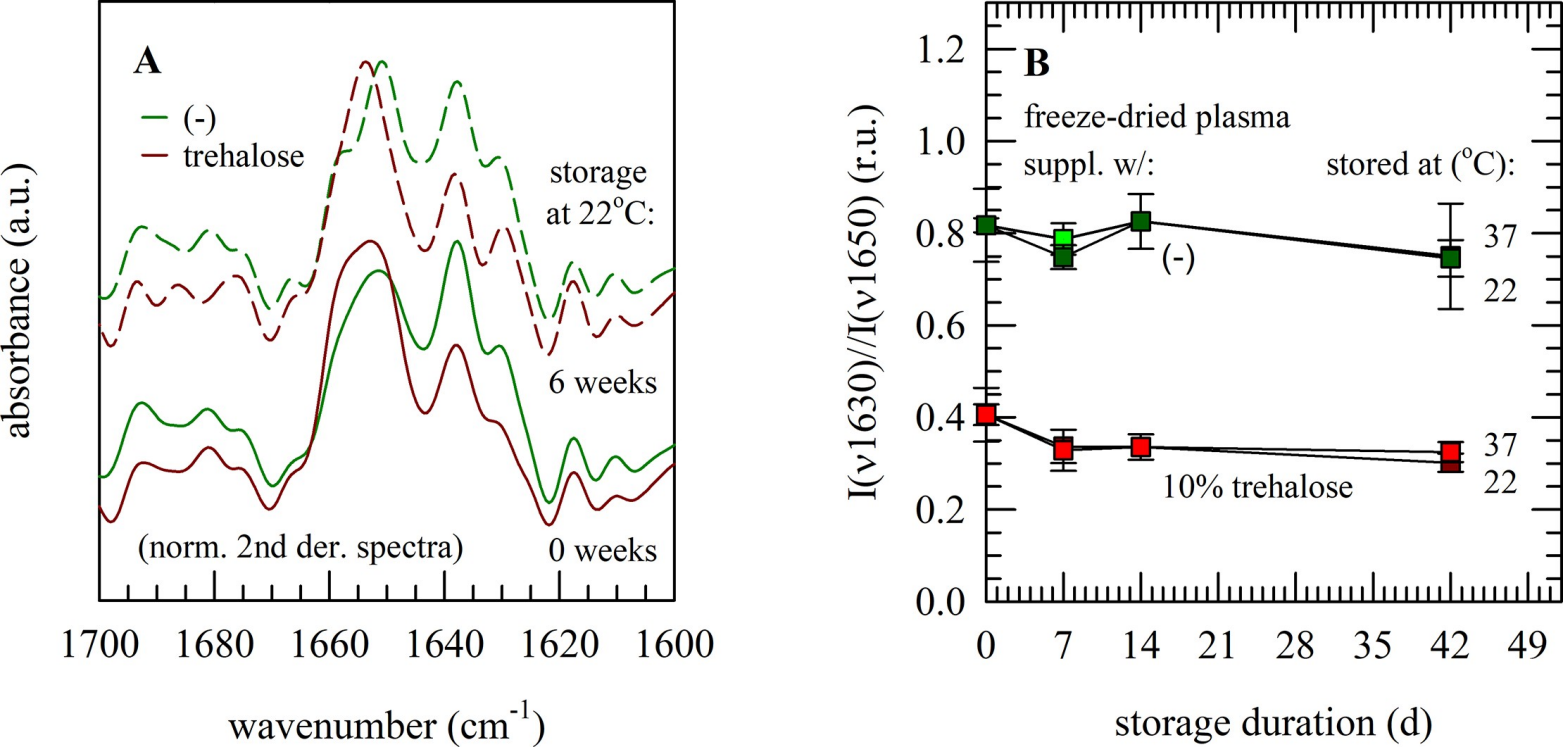
Infrared spectroscopic analysis of freeze-dried plasma during storage. Storage-related changes in infrared spectra of plasma freeze-dried without protectants (green) or with 10% trehalose (red). Spectra were acquired immediately after freeze-drying (solid lines) as well as 6 weeks storage (dashed lines) at 22°C; and normalized second derivative spectra were calculated for the 1700−1600 cm^−1^ region (A). The ratio between the band intensities at ~1630 and ~1650 cm^‒1^, representing respectively α-helical and β-sheet structures, was calculated to reveal changes in the protein secondary structure during storage at 22 or 37°C (B). Mean values ± standard deviations were calculated from three replicates/measurements performed using plasma from three different donors.

Whereas the overall protein secondary structure of plasma shows signs of increased contents of β-sheet structures without protective measures, no further changes were observed during storage. The relative content of β-sheet structures and I(ν1630)/I(ν1650) band ratio remained unaffected (Fig 5B), during storage for 6 weeks (at room temperature as well as 37°C).

### Assessment of oxidative damage in plasma samples during hydrated and dried storage, without and with protective agents added

**Fig 6:**
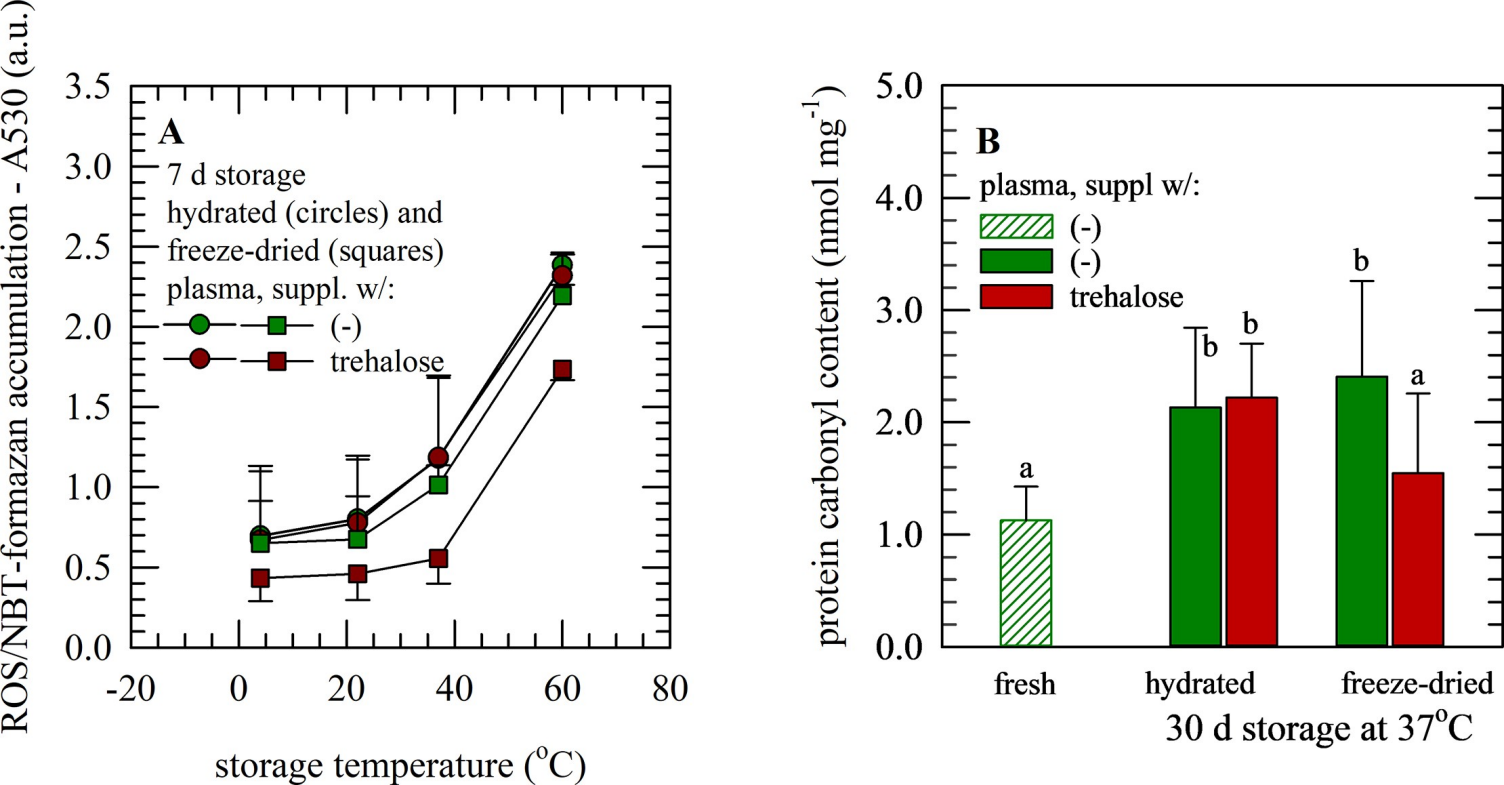
Oxidative damage of freeze-dried plasma during storage. Accumulation of oxidative stress/damage as ROS (A) and protein carbonyl contents (B), in plasma samples during hydrated storage (circles) as well as storage in the freeze-dried state (squares). Plasma without supplements (green) as well as supplemented with 10% trehalose (red), was stored at temperatures ranging from 4−60°C for 7 d (A) or 30 d at 37°C (B). In case of determining plasma carbonyl contents also fresh specimens were analyzed (bars with diagonals) Mean values ± standard deviations are presented for replicate measurements using plasma obtained from three different donors.

Accumulation of ROS, in hydrated as well as dried plasma samples was evaluated during storage using two different approaches. First NBT-formazan formation was monitored, and it was found that plasma freeze-dried with trehalose exhibits a lower amount of ROS and formazan accumulation as compared to samples freeze-dried without protectants, especially for specimens stored at temperatures up to 37°C. ROS accumulation in plasma freeze-dried without protectants appeared similar as in hydrated samples (Fig 6A). A similar trend was seen for protein oxidation and carbonyl contents of plasma samples. Plasma protein carbonyl contents after 30 d storage at 37°C were lowest for freeze-dried samples with trehalose whereas freeze-dried samples without protectants and hydrated samples exhibited higher and similar protein carbonyl contents (Fig 6B). Moreover, plasma protein carbonyl contents for samples subjected to freeze-drying with trehalose and 30 d storage were found to be similar compared to those of plasma not subjected to freeze-drying or storage.

## Discussion

In this study it was found that protein aggregation and conformational changes in human plasma subjected to freeze-drying, can be prevented by adding sugars. The drying-induced increase in plasma turbidity, and concomitant changes in overall protein secondary structure, can almost entirely be prevented using 10% (w/v) glucose, sucrose or trehalose. DSC analysis confirmed that plasma freeze-dried with trehalose is in a glassy state at room temperature. Storage in a glassy state decreases the extent of protein aggregation and oxidative damage versus time. The glass transition temperature of glucose appears to be too low for room temperature storage. Moreover, glucose is a reducing sugar and thus reacts with proteins via Amadori and Maillard reactions **[30]**. These reactions can even occur in dried samples. Trehalose and sucrose have been widely used to stabilize biomolecules in numerous pharmaceutical and medical applications **[31,32]**. Trehalose increases the shelf life of freeze-dried (recombinant) proteins and nucleic acids even if samples are stored at elevated temperature **[33,34,35]**. Albumin is the most abundant plasma protein and combining sugars with albumin increases the Tg and average strength of hydrogen bonding within the glassy matrix compared to that of sugars alone **[36]**. This in turn has been associated with decreased molecular mobility/viscosity, which has beneficial effects during long-term storage (i.e. slowing damaging chemicals reactions).

Turbidity measurements after rehydration appeared a simple method to investigate the effects of freeze-drying on plasma samples. Increased turbidity upon rehydration coincided with increased contents of β-sheet structures in the dried state. Protein aggregation is undesirable if plasma is to be used in medical applications (i.e. transfusion). Also for diagnostic purposes, protein conformational changes may interfere with certain assays. For example, protein/antibody and nucleotide recognition sites may be masked or damaged. Application of ELISA for assessment of IgG contents after freeze-drying and storage, when measured with ELISA, revealed no differences as compared to assessments done on hydrated samples or frozen controls. Its applicability for recognition of specific sites (i.e. relevant for disease diagnostics), however, needs to be determined. The HRP-conjugated protein used here for recognizing IgG also binds aggregated IgG **[37]**. Plasma epitopes are not masked and are still recognized by the HRP-conjugated protein.

It is well known that sugars are able to preserve proteins in a stable glassy state, which is formed upon drying **[38].** Several theories exist that explain stabilization of proteins by sugars **[39].** These include replacement of hydrogen bonds with water surrounding biomolecules, and formation of a highly viscous matrix, which slows down damaging reactions. Among the group of disaccharides, especially trehalose has been implicated for its lyoprotective properties, because it contains a strong glycosidic bond, which is less susceptible to hydrolysis compared to the bond in sucrose **[31,40]**. The Tg of a trehalose, i.e. the temperature below which a protective glassy state is formed, is much higher than that of other disaccharides such as sucrose **[41]**. Moreover, added sugars will remain within the samples, which may interfere with specific diagnostic applications. Therefore, the use of trehalose is preferred over glucose or sucrose, since trehalose is not commonly found in mammalian cells. Interestingly, although mammals do not have a pathway for trehalose biosynthesis, they do possess intestinal and renal thehalase, an enzyme that hydrolyses trehalose into glucose **[42]**.

Moisture uptake by dried samples, which decreases the glass transition temperature, and generation of ROS are major factors determining protein stability in dried specimens **[43]**. We aimed to monitor accumulation of oxidative damage during storage under various conditions by looking at NBT-formazan staining upon reacting with ROS and determining protein carbonyl contents (i.e. protein oxidation products). Although in some cases formazan formation was visible as blue coloration in the dried specimens, additional procedures were needed for quantification. Samples were rehydrated and incubated for a defined duration, which multiplied the pool of endogenous oxidative damage and formazan formation for detection (i.e. proportionally amongst samples). It is shown here that trehalose reduces the formation of ROS products and protein oxidation products during storage, which could possibly be attributed to its scavenging propertie**s [16]** and its glass forming properties reducing biochemical reactions. To further limit oxidative processes during storage, samples could be supplemented with antioxidants such as α-tocopherol and ascorbic acid.

Taken together, protein aggregation in freeze-dried human plasma can be diminished and even prevented, if freeze-drying is done in the presence of sugars. Because of its relatively high Tg, trehalose allows storage at room temperature, and also protects under sub-optimal conditions such as elevated temperatures. Dry biobanking of plasma is an attractive alternative to traditional cryogenic preservation.

## Supporting information

S1 File Minimal data set of all presented figures. (XLSX)
